# Uneven Magnitude of Disparities in Cancer Risks from Air Toxics

**DOI:** 10.3390/ijerph9124365

**Published:** 2012-12-03

**Authors:** Wesley James, Chunrong Jia, Satish Kedia

**Affiliations:** 1 Department of Sociology, University of Memphis, Memphis, TN 38152, USA; Email: wljames1@memphis.edu; 2 School of Public Health, University of Memphis, Memphis, TN 38152, USA; Email: skkedia@memphis.edu

**Keywords:** air toxics, cancer risk, environmental justice, race, income, disparity, Cancer Alley

## Abstract

This study examines race- and income-based disparities in cancer risks from air toxics in Cancer Alley, LA, USA. Risk estimates were obtained from the 2005 National Air Toxics Assessment and socioeconomic and race data from the 2005 American Community Survey, both at the census tract level. Disparities were assessed using spatially weighted ordinary least squares (OLS) regression and quantile regression (QR) for five major air toxics, each with cancer risk greater than 10^−6^. Spatial OLS results showed that disparities in cancer risks were significant: People in low-income tracts bore a cumulative risk 12% more than those in high-income tracts (*p* < 0.05), and those in black-dominant areas 16% more than in white-dominant areas (*p* < 0.01). Formaldehyde and benzene were the two largest contributors to the disparities. Contributions from emission sources to disparities varied by compound. Spatial QR analyses showed that magnitude of disparity became larger at the high end of exposure range, indicating worsened disparity in the poorest and most highly concentrated black areas. Cancer risk of air toxics not only disproportionately affects socioeconomically disadvantaged and racial minority communities, but there is a gradient effect within these groups with poorer and higher minority concentrated segments being more affected than their counterparts. Risk reduction strategies should target emission sources, risk driver chemicals, and especially the disadvantaged neighborhoods.

## 1. Introduction

Differential exposure to air toxics is a serious public health issue in the US. Air toxics, also known as hazardous air pollutants, are a wide spectrum of air pollutants that pose carcinogenic, neurological, and respiratory effects on humans [1,2]. Examples of common air toxics include benzene, a confirmed carcinogen that exists in gasoline vapors and vehicle exhausts [3], formaldehyde, a carcinogenic gas released from home insulation and particle boards [4], and naphthalene, a semi-volatile compound found in moth repellents and produced by incomplete combustion [5]. Human health risks, especially the cancer risk arising from chronic inhalation of low-dose air toxic mixtures, have been identified as a research priority by a number of US regulatory agencies [6,7,8]. Health risks are unevenly distributed due to differential exposure burdens among different segments of the population [9]. There is a copious amount of literature that examines the disparity in exposures to and cancer risks of air toxics [10,11,12,13,14,15]. 

**Figure A1:**
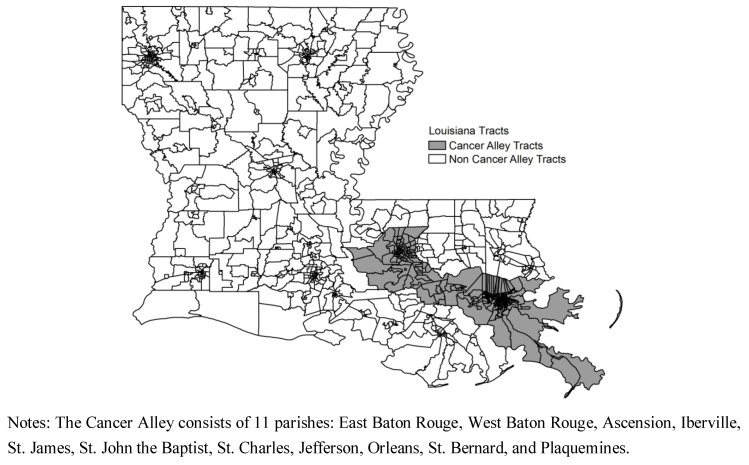
Louisiana and Cancer Alley Tracts.

As the name suggests, “Cancer Alley” serves as a natural test bed for examining disparities in cancer risks from air toxics, not only because of the preponderance of petrochemical industries in this region, but also for its socioeconomic and racial diversity. Cancer Alley stretches approximately 100 miles from Baton Rouge to New Orleans in southeastern Louisiana (Figure A1, see Appendix). The region accounts for approximately 25% of the nation’s petrochemical production, consisting of over 130 plants, refineries, landfills, and factories [16]. Socioeconomic status (SES) data in Cancer Alley reflects low levels of income and high levels of poverty and illiteracy [17]. The racial makeup of Cancer Alley is 55% white and 40% black, compared to state averages of 64% and 32%, and national averages of 75% and 12%, respectively [18]. A total of 79 census tracts in Jefferson, St. John the Baptist, East Baton Rouge, and Orleans Parishes are comprised of at least 90% black residents, and most of these tracts also report exceptionally low household incomes [18]. For the past few decades it is debated whether residents of Cancer Alley experience higher than average rates of morbidity compared to the rest of Louisiana or the nation. Cancer death rates in Cancer Alley are found to be consistent with the average rate for Louisiana [19]. Residents living adjacent to petrochemical plants fail to report any substantial mortality differentials [20,21], meaning that residents at presumably the greatest risk do not report worse outcomes. 

Existing disparities in cancer risks from air toxics have not been well understood for Cancer Alley as well as other regions. Air toxics data are scarce in terms of space and time, in contrast to criteria pollutants that have been routinely monitored by a nationwide network. Since emission information is readily available from EPA’s Toxic Release Inventory (TRI) system, previous air toxics disparity studies focus on the uneven spatial distribution of toxics release sources, which are not directly related to human health. The degree of disparity has mostly been qualified using distance-based geospatial methods [22], which have limitations for assessing risks [23]. In addition, regular statistical methods, e.g., regression analyses and other inferential methods, compute disparities by comparing differences in the mean or the median risks. However, the magnitude of disparities may not be constant over the exposure range encountered. Thus, new techniques are needed to capture the variation in magnitude of disparities.

This research applies spatial quantile regression (QR), in addition to conventional linear regression, to explore income- and race-based disparities in cancer risk among this population. There is a great deal of uncertainty about the health implications of toxic emissions in this region, including possible differential effects across population groups in sub-county geographic locations. Our research examines this issue by investigating income and racial differences in exposure to toxic emissions at the census tract level. It explores the differential distributions of cancer risk caused by ambient air toxics over socioeconomic and race gradients, and proposes measures to reduce risks by identifying major risk contributors and magnitude of disparities.

## 2. Data and Methods

### 2.1. Air Toxics and Socioeconomic Data

Cancer risks from air toxics were obtained from the US Environmental Protection Agency (EPA)’s 2005 National Air Toxics Assessment (NATA) database, which is the latest available nation-wide air toxics exposure information. This database contains concentrations, exposures, and cancer and non-cancer risks for 179 air toxics, as well as their contributing sources, *i.e.*, risks due to exposures from point, non-point, on-road, non-road, secondary, and background sources. NATA is a modeling, not a monitoring program, that estimates ambient concentrations and health risks using a four-step modeling process [24]: (1) compiling outdoor emission sources, including point stationary, non-point stationary, on-road mobile, non-road mobile, secondary, and background sources; (2) modeling ambient concentrations using HEM-3, ASPEN, and CMAQ models; (3) estimating inhalation exposures using the HAPEM5 model that accounts for human time-activity patterns in various microenvironments; (4) characterizing cancer risks due to inhalation exposures. NATA only estimates exposures and risks resulting from outdoor sources, but not those from indoor sources. The lowest spatial scale for estimates is census tract, a geographic subdivision designed to be homogeneous in terms of population characteristics, economic status, and living conditions [25]. Further details of the data sources, methods, models, and assumptions for this dataset can be referred at EPA’s technical report [24]. A model-to-monitor comparison study found a high level of agreement between 2005 NATA and field data collected from over 800 monitoring sites, indicating the general validity of NATA estimates [26]. 

Cancer risk is defined as the probability of contracting cancer given a level and duration of exposure, and it is unitless, with a value between 0 and 1. The probability represents an excess risk that is in addition to any cancer risk borne by a person not exposed to these air toxics. The use of cancer risk normalizes exposure of different air toxics to a comparable measure by accounting for their toxicities. NATA estimates cancer risks for individual air toxics. The cumulative risk is computed as the sum of risks from all carcinogenic chemicals, assuming additivity of cancer risks. A cancer risk is of concern if it exceeds the EPA’s risk benchmark of 1 per million (10^−6^). 

Socioeconomic and racial composition data were obtained from the Census 2000, Summary File 3, available through the US Bureau of the Census [18]. American FactFinder was used to access all detailed tables at the tract, state, and national levels. Demographic and other SES related variables included: population density, median household income in 1,000s, percent of the population that is black, percent of the population below the poverty level, percent of the population aged 65 and older, percent of households that are female headed with children, and percent of the population with less than a high school education. Decennial Census data was used to conduct the initial factor analysis in order to provide full population coverage for a wide range of social and economic variables. Once the factors were determined, all subsequent analyses utilized American Community Survey data averaged 2005–2009 in order to best match the year of population data with that of the NATA estimates. Descriptive statistics of these variables were summarized in Table A1 (see Appendix) at the national, Delta Region Authority (DRA), Louisiana state, and Cancer Alley level (aggregation of 475 census tracts within 11 Parishes) for the comparison purpose.

### 2.2. Data Analysis

The 2005 NATA cancer risk estimates and census data were merged by FIPS county code and census tract number. Descriptive statistics of cancer risks were computed using the county-level data for the nation, DRA, state, and Cancer Alley levels for the comparison purpose. 

SES and race variables are known to be correlated, e.g., minorities often have low household income [27]. Thus, SES and race variables were initially grouped using factor analysis to avoid multicollinearity. Factors were identified based on eigenvalues >1 and factor loadings >0.5, and a Varimax rotation was added to see if a better factor pattern could be obtained. Factor analyses revealed two groups: Group 1 represented the socioeconomic and racial characteristics and Group 2 the population characteristics (Table A2, see Appendix). Hence, tract-level median household income (shortened as “income”) and percent black (as “race”) were selected from Group 1 for the further analyses. Due to the strong correlation between income and race, the two variables were separated in all subsequent analyses to determine SES and racial disparities along these dimensions. Factor 2 was subsequently dropped from the analyses as it did not contribute any additional explanation of socioeconomic disparities in risk exposure beyond that of race and income. 

The disparities of cancer risks were evaluated using geographically weighted regressions, in which income or race was the predictive variable. We first examined the spatial dependence in the data, which were aggregated to a set of geographic units, *i.e.*, census tract here. The univariate Moran’s I tests detected presence of spatial auto-correlation (*p* < 0.05) in risk estimates and socioeconomic and race variables. Thus we used a spatial error model to examine risk as a function of income or race:

Risk = β_0_ + β_1_ Income (or Race) + λWe + µ (1)

where β_0_ = the intercept, β_1_ = regression coefficient of income or race, λ = spatial autoregressive coefficient, W = the spatial weights matrix, e = the random error term in the regression model without the spatial error term, µ = the spatially independent error term. This model is considered more appropriate to correct the bias due to spatial autocorrelation in geographic data [14,28]. The spatial weights matrices were generated using the “makew” function in the McSpatial package in R (ver 2.15.1). Weights were determined using a “ring” method in which neighborhoods were identified by a critical distance of 2 km [29].

Model (1) was run using two regression methods: ordinary least square (OLS) linear regression and quantile regression (QR). In OLS regressions, a coefficient of an income or race variable represents the change in mean risk per change of the SES or racial variable. If this coefficient is significant (*p* < 0.05), then the disparity exists. The value of the coefficient indicates the magnitude of disparity, with a unit of 1 per million risk per $10,000 increase in household income, or 1 per million risk per 10 percentage point increase in percent black. Results from OLS models assume a constant disparity magnitude over the whole Cancer Alley population; however, the magnitude may vary. The uneven disparity magnitude was further examined using QR. QR is a well-developed technique that provides a rather complete picture of dependencies by modeling data with differing variance [30]. QR is similar to OLS linear regression, but extends the least squares estimates of conditional means for a range of models estimating conditional quantile functions [31]. In QR, disparity is evaluated as change in the quantile risk, rather than the mean risk, per change of the independent variable. QR is particularly useful when the rate of change in the conditional quantile risk is expressed by the regression coefficients. Overall, QR provides a fuller analysis of disparities in risks from air toxics. All linear regression analyses were conducted in SAS 9.2 (SAS Institute Inc., Cary, NC, USA).

Spatial regression analyses were performed for 5 volatile air toxics that had their individual mean risks exceeding 1 per million, as well as the cumulative risk from all carcinogenic toxics. Carbon tetrachloride and ethylene oxide, both having mean risks over 1 per million, were not included in disparity analysis (for the reasons see Results). Diesel exhaust, an indicator of exposure from traffic sources, was not included as EPA has not developed a dose-response relationship to estimate inhalation cancer risk. The uses, sources, and target cancers of these compounds are summarized in Table A3. The probability distributions of all risks were symmetric, as ambient concentrations in NATA were estimated using Gaussian dispersion models that assume the concentration distribution to be Gaussian in both the vertical and horizontal directions. Spatial distributions of the total cancer risks by income and race classifications were visualized at the census-tract level in ArcMap 10 (ESRI, Redlands, CA, USA).

## 3. Results

### 3.1. Estimates and Contributors of Cancer Risks

**Figure 1:**
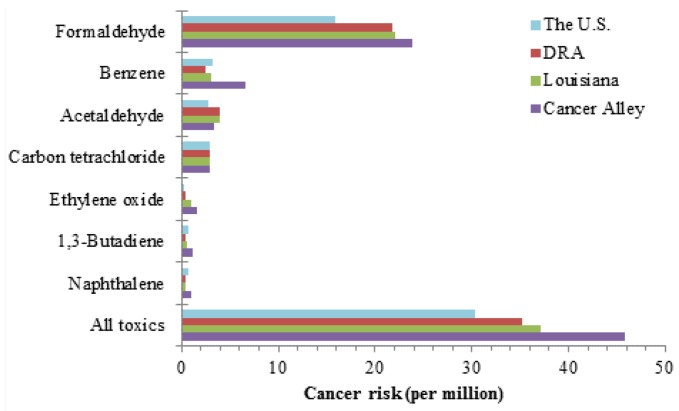
Comparison of mean cancer risks among the US, DRA, Louisiana and Cancer Alley.

In Cancer Alley, the mean cumulative cancer risk was 45.8 per million, meaning that up to 46 individuals out of one million could potentially develop cancer over a lifetime exposure to all carcinogenic air toxics in ambient air. This risk level was significantly higher (*p* < 0.05) than 30.3, 35.3, and 37.1 per million in the US, DRA, and Louisiana, respectively (Figure 1 and Table A4). Seven individual compounds had mean risks exceeding the benchmark level: formaldehyde, benzene, acetaldehyde, carbon tetrachloride, ethylene oxide, 1,3-butadiene, and naphthalene. Their mean risks ranged from 1.0 to 23.8 per million, and they were considered “risk drivers” among all the air toxics. The individual risks were also higher than the corresponding national, DRA, and state levels. These facts indicate that the population in Cancer Alley, as a whole, has higher exposure burden and cancer risks. 

**Figure 2:**
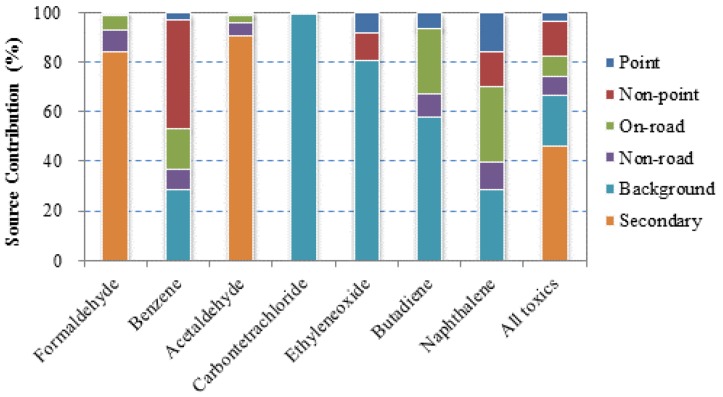
Contributions of various sources to cancer risks in Cancer Alley.

Source contributions to the total cancer risks varied greatly between compounds (Figure 2). Carbon tetrachloride has no indoor and outdoor source, and is present in the environment at a stable concentration. Ethylene oxide is a highly reactive chemical, and its concentrations are also low and relatively uniform. These two chemicals mainly displayed background concentrations, and thus, they were excluded from further analysis of disparities. Formaldehyde and acetaldehyde are two aldehydes that are formed through atmospheric reactions [32], so their total risks were largely attributed to secondary sources. Other compounds had relatively high contributions from emission sources: benzene risks were dominated by point and non-point stationary sources, and 1,3-butadiene and naphthalene risks by on-road mobile sources. Secondary and background sources contributed to the largest portions (55.1% and 19.1%, respectively) of the cumulative risk; however, risks from these sources were only slightly higher in Cancer Alley compared to the national levels. Hence, it was emission sources, including point, non-point, on-road, and non-road sources, that made the significant difference. Among the four emission sources, point sources were the largest contributor (9.7%), suggesting the concerns of emissions from major industrial facilities in Cancer Alley. 

### 3.2. Socioeconomic Disparities in Cancer Risk

**Figure A2:**
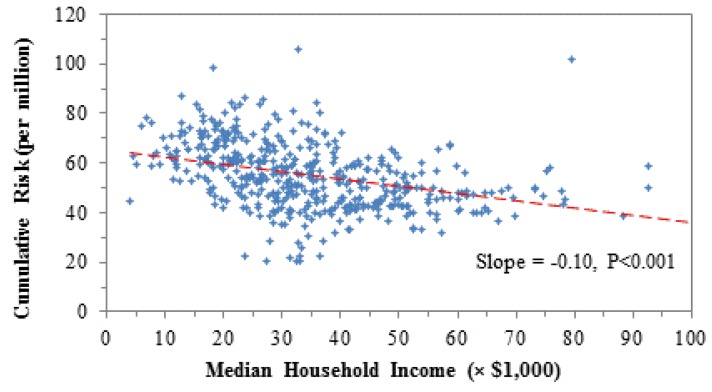
Relationship between the cumulative risk from all toxics and household income.

Cancer risks increased with decreasing household income (Table 1), accounting for spatial autocorrelation. This relationship could be demonstrated using the cumulative risk of all toxics as an example in Figure A2. The coefficient was −0.98 units, meaning an increase of 1 per million risk every $10,000 decrease in tract-averaged household income. In other words, a decrease of $10,000 in income was equivalent to exposure to one extra risk driver, e.g., naphthalene or 1,3-butadiene (Figure 1). The resulting health effects are that one additional person is likely to develop cancer out of a one million population. Compound-wise, the five risk drivers contributed 90% of the total magnitude of SES disparity. Formaldehyde and benzene were the two largest contributors, accounting for 39% and 36% of the total disparity magnitude, respectively. Source-wise, the four emission sources contributed 79% of the SES disparity, and on-road vehicular emissions were the largest, accounting for 48%. Therefore, eliminating SES disparity in air toxics exposure should focus on risk drivers and on-road emissions.

**Table 1:** Effects of household income and race on cancer risks.

VOCs	Income ^1^	95% C.I.		Race ^2^	95% C.I. ^3^	
Formaldehyde	−0.38	(−0.60,	−0.17)	0.42	(0.28,	0.55)
Benzene	−0.36	(−0.49,	−0.22)	0.50	(0.42,	0.58)
Acetaldehyde	−0.03	(−0.04,	−0.01)	0.02	(0.01,	0.04)
1,3-Butadiene	−0.06	(−0.09,	−0.04)	0.05	(0.04,	0.07)
Naphthalene	−0.05	(−0.08,	−0.03)	0.03	(0.02,	0.05)
All toxics	−0.98	(−1.36,	−0.60)	1.12	(0.88,	1.36)

*Notes*: ^1^ A slope coefficient represents decrease of cancer risk (unit: 1 per million) every $10,000 of increase in income. Slopes are highlighted if they are significantly different (*p* < 0.05) from 0.

^2^ A slope coefficient represents increase of cancer risk (unit: 1 per million) every 10 percentage point increase in percent of the black population.

^3^ C.I. = Confidence Interval.

The disparity in cancer risks remained significant if household income was categorized. According to Thompson and Hickey [33], household income could be categorized as low (<$25,400, bottom 20%), medium ($25,500–$41,500, middle 32%), and high (≥$41,600, top 48%). Low-income tracts bore a cumulative risk 12% more than high-income tracts (*p* < 0.05), and 7% more than medium-income tracts (*p* < 0.05). The disparity magnitude was greatest for benzene, naphthalene and 1,3-butadiene, all vehicle-related chemicals. Income classification clearly demonstrated substantial differences in exposure to air toxics and the associated cancer risks in Cancer Alley. 

**Figure 3:**
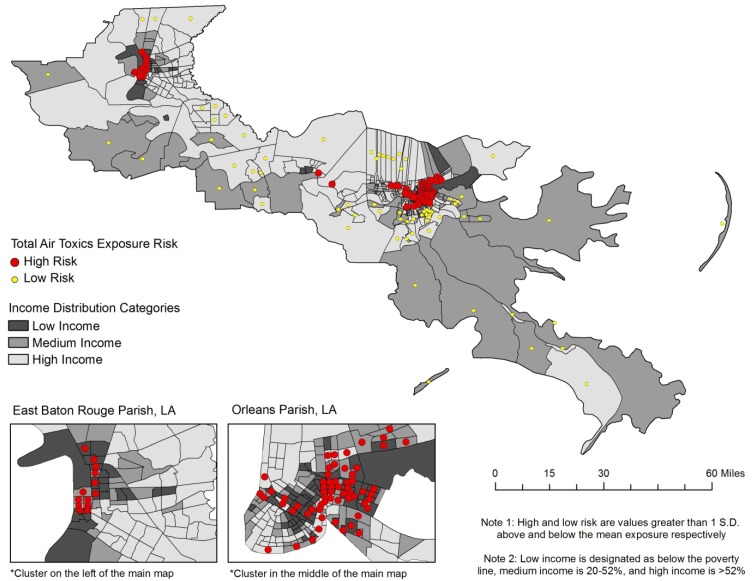
The cumulative risk from all toxics by income in Cancer Alley tracts.

The effect of household income on cancer risk can be visualized in Figure 3. The highest risks (red dots, risk greater than 1 standard deviation above the mean) are clustered in the lowest income tracts (dark gray), most notably in East Baton Rouge and Orleans Parishes. In East Baton Rouge Parish there are 13 tracts defined as high risk, and seven of them are classified in the lowest income category and only one is in the high income category. In Orleans Parish, 62 of the 181 tracts are classified as high risk, and most of these are also low to medium income categories. Overall, high risk tracts in Orleans Parish have a combined median household income of $29,600 per year. The remaining tracts in Orleans Parish have a combined median household income of approximately $42,700 per year. In East Baton Rouge Parish the income differential is even greater. High risk tracts in East Baton Rouge Parish have a median household income of $27,000 per year, compared to $60,100 for low risk tracts, and $46,600 for the remaining tracts. These statistics further illustrate that the highest risk of cancer is disproportionately located in the poorest tracts while the lowest risk of cancer is found among the higher income tracts. 

### 3.3. Racial Disparities

**Figure A3:**
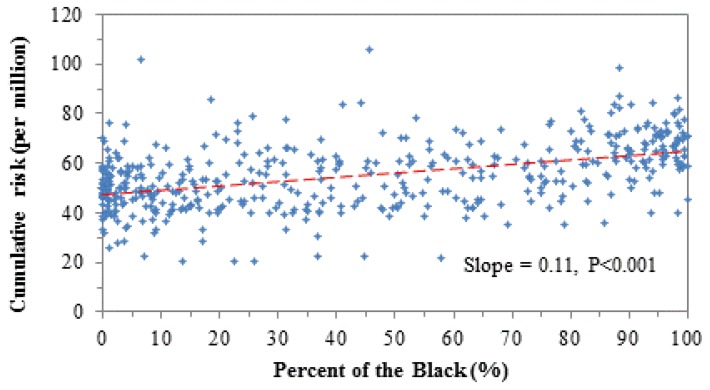
Relationship between the cumulative risk from all toxics and percent of the population that is black.

Linear models using race as an independent variable gave results similar to the models using income, and revealed disparities in terms of race. Race effects on cancer risk are given for individual toxics and all toxics in Table 1, and are demonstrated using the cumulative risk from all toxics in Figure A3 (see Appendix). As the percentage of black residents increases, so does the cancer risk. The coefficient of the cumulative risk was 1.12 units, meaning an increase of 1.12 per million risk getting cancer for every 10 percentage point increase in the percent black of the population. This is the equivalent of an additional risk driver being introduced into the environment, e.g., 1,3-butadiene or naphthalene (Figure 1). The five risk drivers contributed to 92% of the total racial disparity, and benzene and formaldehyde were the two largest contributors, contributing 37% and 45%, respectively. When examining sources, point, non-point, on-road, and non-road emission sources contributed 73% of the total racial disparity, and the largest contributors were on-road and non-point sources, with contributions of 37% and 30%, respectively. 

Again, racial disparity was significant if tracts were categorized in terms of race. Tracts were categorized as: black-dominant (>75% black), mixed race (25–75% black), and white-dominant (≤25% black) [34]. The cumulative risk from all toxics increased in black dominant areas by 16% compared to that in white dominant areas (*p* < 0.0001), and by 5% compared to mixed areas (*p* < 0.01). Risk differences between race categorized areas did not always follow results from those obtained in OLS regressions, indicating inconsistent disparity magnitude over the risk range. This finding necessitated additional analysis of how magnitude of disparity was changing with risk levels, *i.e.*, QRs that are presented later. 

**Figure 4:**
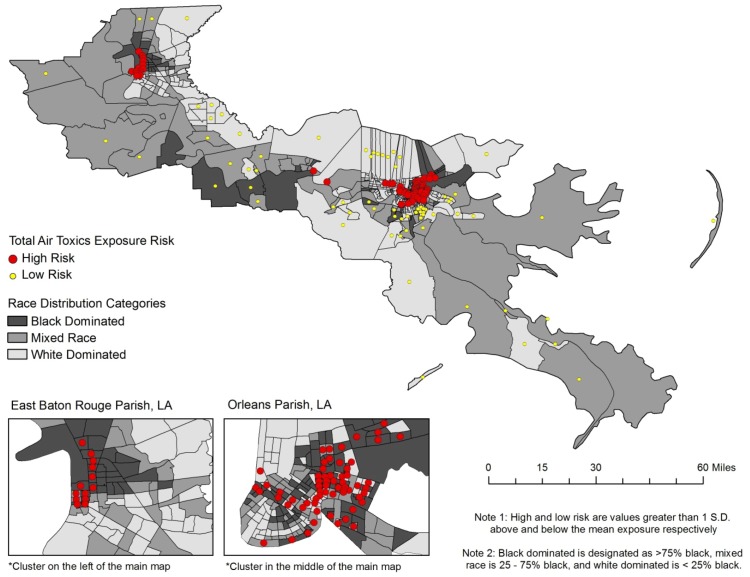
The cumulative risk from all toxics by race in Cancer Alley tracts.

Spatial patterns of risk clusters and the racial composition of each Cancer Alley tract is given in Figure 4, providing further evidence of racial disparities in cancer risk. The highest risks (red dots) are clustered in the tracts with the highest proportion of black residents (dark gray), primarily in East Baton Rouge and Orleans Parishes. Eleven of the thirteen high risk tracts in East Baton Rouge Parish have at least 75% black population, and most are well above 90%. As a collective, the high risk tracts in this parish are 84% black. Alternatively, the low risk tracts in East Baton Rouge Parish are a combined 29% black, and the remaining tracts are 46% black. In Orleans Parish the findings are not as dramatic, but remain disproportionate for the higher percentage black tracts. There are 62 high risk tracts, 35 of which are black dominant (75% black or greater), and 17 are white dominant. There is only one low risk tract in Orleans Parish and it has no black population. Overall, high risk tracts in Orleans Parish are on average 60% black, compared to approximately 50% black in the remainder of tracts. A significant cluster of low risk tracts is found in three parishes adjacent to Orleans; Jefferson, Plaquemines, and St. Bernard. Nearly all of these tracts range from 75% white to more than 90% white. This provides clear evidence for racial disparities in cancer risk throughout Cancer Alley.

### 3.4. Uneven Magnitude of Disparity

**Figure 5:**
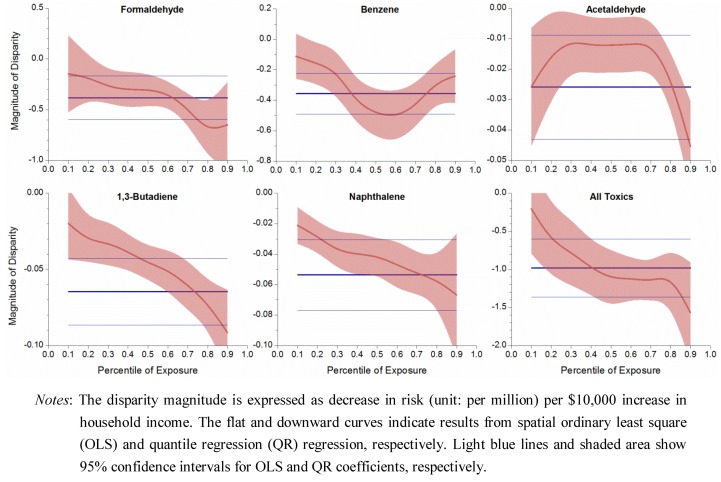
Uneven magnitude of socioeconomic disparity over exposure range.

Spatial quantile regression results showed that disparity magnitude varied along the exposure or risk range, as opposed to the constant magnitude obtained in OLS regression. Magnitude of SES disparity was plotted against percentile exposure, as displayed in Figure 5. 

The coefficient from QLS regression, displayed as a flat line, indicated a constant disparity magnitude. The negative value meant that risk decreased in higher income households and could inform the disparity magnitude. Coefficients from QR regressions generally showed a downward curve, indicating that disparity magnitude increased at high risk end. For the total risk from all toxics, the disparity magnitude was −0.2 unit at the 10th percentile exposure, but −1.6 units at the 90th percentile exposure, which could be translated into a drop of 0.2 per million cancer risk per $10,000 income increase at the low exposure tracts, but a larger drop of 1.6 per million risk per $10,000 income increase at the high exposure tracts. Benzene was the only exception, in which its disparity magnitude was the largest in the middle range of exposure. As low-income tracts had high exposure and risk levels, QR analyses illustrated that, not only income disparity existed for the cumulative risk, but also its magnitude became larger in the low-income neighborhoods. 

**Figure 6:**
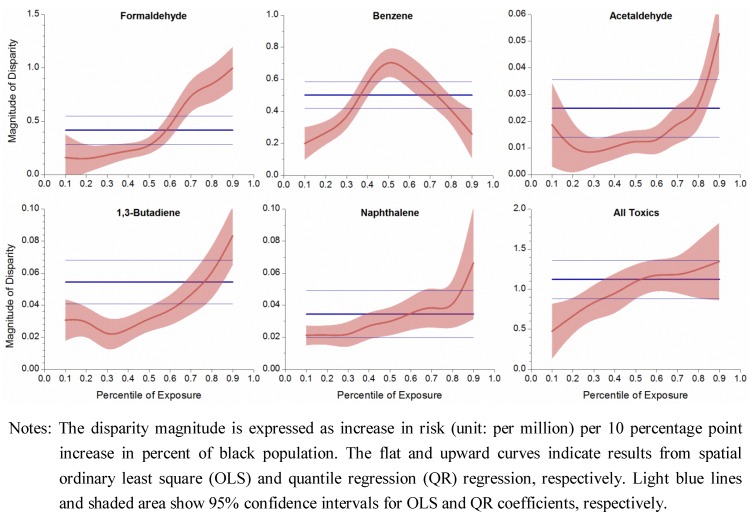
Uneven magnitude of racial disparity over exposure range.

The QR curves in Figure 6 were nearly mirror images of those in Figure 5, as household income and percent of black are highly negatively correlated. The QLS coefficients, as flat lines in Figure 6, indicated constant racial disparities for individual compounds and all toxics, and the positive value meant a positive relationship between risk level and percent of black population. 

QR coefficients displayed an upward trend with increasing exposure quantiles, meaning that disparity magnitude increased at high end risk, *i.e.*, in black dominant areas. For example, the disparity magnitude was 0.5, 1.1, and 1.3 units at 10th, 50th, and 90th percentiles, respectively, for the total risk from all toxics, which means that as the percent of the black population increased by 10 percentage points, cancer risk increased by 0.5, 1.1, and 1.3 per million at white-dominant, mixed-race and black-dominant areas, respectively. Overall, QR results showed that racial disparity in cancer risk from air toxics worsened as minority concentration increased. 

The risk magnitudes of the five compounds and all toxics combined showed strong patterns. Some ubiquitous chemicals, e.g., carbon tetrachloride, would have general constant QR coefficients over the exposure range with slight fluctuations, a situation called “location shift” in which the disparity magnitude is relatively uniform. The first group shows that QR provides information in addition to the conventional “average” effects; the second group has comparable results in the case of a location shift. Combining OLS and QR results reflects that cancer risk disparity not only exists among economically stratified and racial groups, but its degree exacerbates in poorer and black-dominant areas. 

## 4. Discussion

### 4.1. Explanations of Risk Disparities in Cancer Alley

Health disparities are often the result of inequalities from the social, physical, and built environment [35]; clearly those factors are evident in Cancer Alley. Literature abounds a variety of explanations for the existence of these disparities, both at the macro and micro levels. Environmental justice literature delves into the inequitable environmental burdens experienced by minority and low income communities [36,37]. A national survey reveals that Hispanics and blacks experience much higher personal exposure to aromatic air toxics [38], as evident in our research. From a macro standpoint, environmental injustices are a fundamental outcome of our social structure where powerless communities are inevitably exploited by a more powerful entity, such as a corporation or an industry [39]. The petrochemical industry in Cancer Alley began with the opening of a Standard Oil refinery in Baton Rouge in 1908, and ballooned to more than 300 facilities in the following century due to federal investment into the region, tax exemptions, and liberally applied water discharge permits in communities along the Mississippi River [40]. Since 1997, huge toxic releases have been permitted in the Cancer Alley region, spilling more than 140 million pounds of chemicals into the environment and forever changing the landscape of the industry in southeastern Louisiana [40]. These factories, and consequently chemical emissions, were in proximity to many low income and minority communities; as are roadways of high traffic volume. Residential exposure to traffic related pollution is shown to increase mortality risk (heart and lung ailments primarily) [41], and considering the high proportion of low income and minority residents in the urban tracts of Cancer Alley in East Baton Rouge and Orleans Parishes, this has the potential for disproportionate effects on these individuals who primarily inhabit the inner cities. The production and reproduction of inequality is a process that includes negotiation and conflict among several different parties struggling for resources within a political economy, including corporate executives, government officials, neighborhood residents, community activists, *etc.* [42]. Similar social science work describes the unnecessary and unjustified privilege to the environment by humans that creates unjustified toxic emissions and waste; a socially constructed phenomenon [43]. That is, society has generally accepted the misuse of environmental resources for the benefit of a few [43]. This further illustrates the social nature of the power structure that assists in creating risk disparities in Cancer Alley and environmental justice issues in general.

Micro level theories also provide similar explanations for social inequality. It is common for companies to locate their plants and factories in disadvantaged areas because the residents are politically powerless and do not have the organization or capital to fight back [36,44]. Similarly, the lack of political power also means that there are no advocates or lobbyists representing them at the national level [36]. Another theory is that minority communities are more intimately engaged with other social issues, such as crime, drugs, and poverty [36], *i.e.*, environmental issues takes a backseat to these other pressing issues. Lastly, because of economic, educational, and social barriers, residents of poor and minority communities cannot easily react by relocating. The combination of these factors leads to a double jeopardy, or even a multiple jeopardy situation [45], in which the disadvantaged status of minorities has a cumulative negative effect on their health. Specifically, the negative health effects for a person who is poor and of minority status may be multiplicative. Thus, the concept of double jeopardy is easily applied to race and income disparities investigated in this research, which is a likely cause of the increasing magnitude of disparity in the poorest and minority concentrated areas in this study. As referenced in Figure 3 and Figure 4, the poorest and most black concentrated tracts are often the same tracts, lending credibility to the double jeopardy hypothesis.

### 4.2. Strategies to Eliminate Disparity in Air Toxics Exposure

As eliminating health disparities is a priority of Healthy People 2010 [46], our research informs the need to eliminate health disparities using an environmental justice framework to identify excessive cancer risk in poor and minority concentrated areas due to disproportional exposure to harmful toxic emissions. There are important policy implications of these findings in terms of strategies to reduce or eliminate risk disparities. The strategies to reduce disparities are threefold: (1) control emissions of risk drivers, (2) focus on emission sources including major facilities and mobile sources, and (3) prioritize low income and black dominant areas. 

Focus should be placed on controlling the emissions of risk drivers. Income disparities in cancer risk are largely the product of formaldehyde and benzene emissions. Butadiene and naphthalene are large contributors to racial disparities in cancer risk. These emissions are from point and non-point industrial sources and on-road and non-road vehicular sources, with on-road emissions being the largest contributor to both types of disparities. Each of these compounds are emitted from vehicular exhaust, among other sources, which indicates that low income and predominantly black neighborhoods are located in closer proximity to high traffic areas and/or industrial sites emitting these compounds. The elimination of disparity should target the control of on-road sources such as vehicular emissions, as well as controlling non-road emissions from major industrial facilities. The EPA’s Office of the Inspector General reports that the US needs a national air toxics monitoring network. Many states and communities do not have an adequate monitoring system; this would be a very useful solution to Cancer Alley and other communities around the country that bear high air toxics risks. 

An additional recommendation to reduce disparities is the meaningful involvement of citizens in environmental policy [13]. Strong grassroots campaigns have the potential to influence policy at a governmental level to enforce stricter regulatory framework. Historically, there is a divide between citizens and policy makers in developing environmental and health policies which leads to a disconnect between the interests of various stakeholders operating at different levels (local, state, and federal). It would be critical to work together in sharing information, analyzing risk, and problem solving between civic environmentalists and local, state, and federal decision makers in collaborative manner [47]. Similarly, sub-state government has the ability to control the siting of new facilities [48], and they should make decisions only after careful consideration of the community’s interests via local grassroots initiatives, and knowledge of the health consequences to the groups that have historically been negatively affected by such decisions. For these reasons, the interests of the poor and minority communities should be a priority in the decision making process, as there is sufficient evidence that they are the most vulnerable. In addition, as others have advocated [11], we recommend as an additional priority to educate minority groups about the health implications of living in close proximity to toxic sites through outreach programs and community wide education campaigns.

Lastly, although control of emission sources in disadvantaged areas may reduce or eliminate the environmental disparities, it should be cautioned that it may not completely reduce cancer risks per se. This is especially the case for toxics originating primarily from indoor sources, e.g., formaldehyde and p-dichlorobenzene. Numerous exposure studies have concluded that human exposure is primarily from indoor sources, and the portion from outdoor sources is relatively small. Therefore, it is not clear that lower cancer risk will result in Cancer Alley by reducing the outdoor contribution to air pollution. However, this does not diminish the importance of controlling for outdoor risk drivers.

### 4.3. Limitations

Data restrictions constitute the primary limitation of this research. Although NATA estimates generally agree with the monitoring results at the national level, the concordance could be site specific. The normally distributed modeled ambient concentrations do not reflect the typical right-skewed distributions of measured exposure, e.g., log-normal or exponential distributions, as seen in numerous exposure studies. Cancer risks in NATA reflected risks from exposure to outdoor sources, which represented only a small portion of the actual exposure, given people’s majority of time spent indoors and typically higher indoor concentrations [49]. Risk estimates were obtained by a conservative approach in which a 95% upper confidence limit (UCL) of the mean exposure was used for risk estimation [50]. The regression analyses could not identify specific “hot spots”, as warned by EPA, but rather documented the uneven distribution of air toxics at large geographic scale. Reliable estimates for intercensal years are available through the American Community Survey in five year average estimates due to the small population size of many of these geographic units. Although the population data are useful and reliable, there are no lifestyle and behavioral indicators, such as smoking, alcohol use, diet, and exercise, available at this level of analysis. These known cancer determinants, if included in the models, might overwhelm the potential risk posed by air toxics included in the study. Thus, the cancer risk in this study reflected only environmental exposure, and did not take toxicological, epidemiologic, and behavioral factors into account.

## 5. Conclusions

Cancer Alley, Louisiana, USA, is characterized by a high proportion of black residents, low socioeconomic indicators, and a concentration of toxic emitting factories. The population is disproportionately exposed to the major air toxics compared to three relevant reference groups, the US, DRA, and the state of Louisiana, making it a region of excessively high cancer risk. Our findings suggest that there is strong evidence pointing to disparities in cancer risk based on income and race in Cancer Alley. Cancer risk increases by 12–16% in low-income/black-dominant tracts compared to high-income/white tracts. Disparities are caused by major risk drivers including benzene, formaldehyde, 1,3-butadiene, and naphthalene, and emission sources including on-road, non-road, point, and non-point sources. Last, and most importantly, results from spatial quantile regression analyses reveal that the magnitude of disparities is heightened in the tracts with poorer people and higher concentrations of blacks, suggesting the need to focus on risk drivers, primary emission sources, and low-income or black dominant areas to eliminate disparities more effectively. 

## Appendix

**Table A1:** Descriptive statistics of socioeconomic and racial variables at the national, Delta Region Authority (DRA), Louisiana state and Cancer Alley levels.

Socioeconomic and Racial Variables	The US *	DRA *	Louisiana *	Cancer Alley
Total Population	279,731,048	8,035,514	2,778,118	1,690,858
Population Density	73.9	52.01	63.2	215.1
Median Household Income (in 1,000s)	35.4	30.0	28.7	37.0
Percent in Poverty	12.0	18.6	19.3	18.6
Percent Black	12.1	29.8	27.5	40.1
Percent Age 65+	12.4	13.1	11.9	11.0
Percent Female Headed Household	7.1	9.2	9.2	10.6
Percent < High School Degree	19.4	27.2	27.2	21.8

***** Statistics are generated for the total number of Parishes minus the 11 Cancer Alley Parishes.

**Table A2:** Groupings of socioeconomic and racial variables determined by factor analysis.

Socioeconomic and racial variables		Initial			Rotated	
		Factor1	Factor2		Factor1	Factor2
	Total population	–0.32	**0.78 **		–0.29	– **0.79 **
	Population density	**0.57 **	–0.22		**0.56 **	0.24
	Median household income	– **0.86 **	0.06		– **0.86 **	–0.09
	Poverty percent	**0.93 **	0.04		**0.93 **	–0.01
	Percent of the black	**0.86 **	0.17		**0.86 **	–0.14
	Percent of the population age > 65	–0.22	– **0.83 **		–0.25	**0.82 **
	Percent of female headed household	**0.93 **	0.03		**0.93 **	0.00
	Percent of less than high school degree	**0.83 **	0.02		**0.83 **	0.00

**Table A3:** Risk drivers in Cancer Alley and their sources, cancers of concern and cancer classification.

Air Toxics	CAS No.	Outdoor Sources ^1^	Cancer of Concern ^2^	EPA Cancer Classification ^2^
Formaldehyde	50-00-0	Combustion, oxidation of methane, vehicular exhausts, emissions from resins in particle board.	Squamous cell carcinoma	B1, probable human carcinogen
Benzene	71-43-2	Tobacco smoke, vehicle service stations, motor vehicle exhaust, industrial emissions	Acute myeloid leukemia	A, known human carcinogen
Acetaldehyde	75-07-0	Production of perfumes, polyester resins, and basic dyes, fruit and fish preservative, flavoring agent, solvent in the rubber, tanning, and paper industries	Squamous cell carcinoma	B2, probable human carcinogen
Carbon tetrachloride	56-23-5	Drinking water, industrial emissions	Possible liver cancer, lymphatic leukemia, non-Hodgkin's lymphoma	Carcinogenicity is undergoing reassessment
Ethyleneoxide	75-21-8	Sterilize medical equipment and supplies, fumigant to spray agricultural products	Leukemia, stomach cancer, pacreatic cancer, Hodgkin's disease	B1, bordering on B2, limitations in human carcinogenic evidence
1,3-Butadiene	106-99-0	Emissions from the production of rubber, plastics, and resins, vehicle engine exhaust, smoke from fires, cigarette smoke	Hemato-lymphopoietic, stomach, and respiratory cancer	A, known human carcinogen
Naphthalene	91-20-3	Burning of wood and fossil fuels, industrial discharges, automobile exhaust, cigarette smoke, moth repellants, asphalt emissions	Pulmonary alveolar proteinosis	C, possible human carcinogen

Sources:

^1^ The Agency for Toxic Substances and Disease Registry (ATSDR)’s toxicological profiles at http://www.atsdr.cdc.gov/toxprofiles/index.asp.

^2^ The US Environmental Protection Agency (EPA)’s Integrated Risk Information System (IRIS) at http://www.epa.gov/IRIS/.

**Table A4:** Descriptive statistics of cancer risks (×10^−6^, *i.e.*, 1 per million) contributed by different sources and the top 10 air toxics. DRA = Delta Region Authority. P90 = the 90th percentile. Individual compounds only included volatile organic compounds (VOCs).

Sources / VOCs	The U.S.				DRA Area*				Louisiana*				Cancer Alley		
	(n = 3,276)				(n = 241)				(n = 53)				(n = 11)		
	Ave	Med	P90		Ave	Med	P90		Ave	Med	P90		Ave	Med	P90
**Sources**															
Point	0.55	0.17	1.34		0.57	0.23	1.09		0.55	0.26	1.19		2.81	2.14	5.83
Non-point	2.73	1.74	6.06		1.68	1.38	2.94		1.88	1.51	3.07		3.99	2.87	5.26
On-road	1.80	0.76	4.44		0.89	0.59	1.71		1.02	0.73	2.01		2.68	1.88	5.82
Non-road	0.71	0.36	1.63		0.40	0.30	0.75		0.60	0.44	1.19		2.97	2.08	4.84
Background	7.20	6.81	10.6		6.81	6.51	8.5		7.96	7.97	9.4		9.36	8.87	11.2
Secondary	17.4	16.6	26.8		24.9	24.9	29.6		25.1	25.4	30.1		24.0	25.6	27.0
Total	30.3	29.1	44.8		35.3	34.6	41.0		37.1	35.9	43.1		45.8	46.4	53.2
**VOCs**															
Formaldehyde	15.9	15.2	24.6		21.8	21.7	26.3		22.1	22.1	26.1		23.8	24.4	28.4
Benzene	3.26	2.86	6.19		2.48	2.16	3.93		3.09	2.81	4.70		6.62	5.91	8.57
Acetaldehyde	2.79	2.66	4.27		3.98	3.98	4.72		3.93	4.06	4.74		3.41	3.36	4.12
Carbon tetrachloride	2.85	2.85	2.87		2.86	2.86	2.87		2.85	2.86	2.87		2.87	2.87	2.88
Ethylene oxide	0.32	0.23	0.69		0.44	0.31	0.96		1.04	1.05	1.45		1.51	1.44	1.83
1,3-Butadiene	0.63	0.45	1.41		0.39	0.34	0.68		0.50	0.45	1.00		1.13	1.14	1.70
Naphthalene	0.63	0.39	1.42		0.36	0.31	0.63		0.39	0.37	0.63		1.03	1.07	1.46

* Excluding counties in the Cancer Alley.
